# PRRSV-2 viral load in critical non-lymphoid tissues is associated with late gestation fetal compromise

**DOI:** 10.3389/fmicb.2024.1352315

**Published:** 2024-02-08

**Authors:** K. Rudy, D. Jeon, A. A. Smith, J. C. S. Harding, J. A. Pasternak

**Affiliations:** ^1^Department of Animal Sciences, Purdue University, West Lafayette, IN, United States; ^2^Department of Large Animal Clinical Sciences, Western College of Veterinary Medicine, University of Saskatchewan, Saskatoon, SK, Canada

**Keywords:** PRRSV, fetal, immune response, non-lymphoid, *in utero* infection, Nidovirus, meconium staining

## Abstract

The impact of late gestation PRRSV-2 infection is highly variable within a litter, with a subset of fetuses displaying varying degrees of compromise following infection while others remain viable despite significant systemic viral load. To understand the underlying cause of this variation, we examined the susceptibility, distribution and impact of viral infection within non-lymphoid tissues. Samples of brain, heart, kidney, liver, lung, and skeletal muscle were obtained from fetuses of pregnant gilts at gestation day 86, and the presence and distribution of CD163+ cells within each tissue evaluated via immunohistofluorescence. Equivalent samples were collected from phenotypic extremes representing resistant, resilient and susceptible fetuses at 21 days following infection of pregnant gilts with PRRSV-2 at day 86 of gestation. Viral load and its impact in each tissue was evaluated by a combination of qPCR, *in vitro* viral recovery, and local expression of IFNG and CD163. Resting populations of CD163+ cells were observed in all six non-lymphoid tissues from healthy day 86 fetuses, though the apparent density and the morphology of positive cells varied between tissue. Viral RNA was detected in all six tissues derived from fetuses previously classified as highly infected, and infectious viral particles successfully recovered. Significantly more viral RNA was detected in heart, brain, lung and skeletal muscle of susceptible fetuses, relative to their viable counterparts. Infection was associated with an increase in the expression of CD163 in brain, kidney and lung. In addition, the presence of virus in each tissue coincided with a significant upregulation in the expression of IFNG, but the scale of this response was not associated with fetal susceptibility. Thus, PRRSV-2 is widely distributed across these susceptible non-lymphoid fetal tissues, and fetal outcome is associated with local viral load in critical fetal organs.

## 1 Introduction

Porcine reproductive and respiratory syndrome virus (PRRSV) is a highly transmissible, single-stranded RNA virus, originally associated with its reproductive consequences in the early 1990’s (Terpstra et al., 1991). Though initially detected in specific geographic regions, the virus now has near global distribution, with endemic and emerging strains found in nearly every major pork producing country. Within the American swine herd, PRRSV was last estimated to cause annual losses in excess of $660 million, 45% of which is incurred by the breeding herd (Holtkamp et al., 2013). Unlike postnatally infected pigs which experience respiratory symptoms and reduced growth performance (Van Reeth et al., 2001; Pasternak et al., 2021), most strains cause limited respiratory symptoms in the infected dams (Ladinig et al., 2015). Thus, the primary economic impact of PRRSV within the breeding herd results from reproductive consequences including an increase in stillbirths, *in utero* death, and abortions. The fetal outcomes following maternal PRRSV infection vary substantially within a litter, with the most susceptible population found dead or autolyzed *in utero* (Benson et al., 2002). An additional portion of the live fetal population is found meconium stained, which is recognized as an initial marker of severe fetal stress and is the first indicator of fetal compromise (Harding et al., 2017). Those pigs that do survive to term are born weak, often congenitally infected and more susceptible to other pathogens (Feng et al., 2001).

PRRSV is able to cross the late gestation porcine placenta, via an as yet unidentified mechanism, and thereby productively infect a subset of fetuses (Kranker et al., 1998). A pair of early studies surveyed viral load across a range of fetal tissues including heart, liver, lung, spleen, tonsils, thymus and umbilical cord, following late gestation maternal challenge (Benson et al., 2002; Rowland, 2010). Virus was, at minimum, sporadically detected in all these tissues, however, the relative frequency of viral recovery led to the conclusion that the fetal thymus was the primary site of viral infection (Rowland, 2010). Since this seminal finding, thymic viral load has been used in conjunction with serum and placenta to categorize infection status in studies designed to elucidate the physiological impact and mechanism of fetal compromise. Serial studies following infection at a fixed time point have shown an orderly progression of infection beginning with the placenta, followed by fetal serum, and then finally the fetal thymus (Malgarin et al., 2019). However, subsequent studies demonstrated that a fetal immune response is only initiated after significant virus has accumulated within the thymus (Van Goor et al., 2020). It was initially hypothesized that the fetal immune response may represent a double-edged sword, combating viral progression, while at the same time compromising fetal development and survival (Rowland, 2010). However, there is limited evidence that the scale of this inflammatory cytokine response plays a role in fetal compromise (Pasternak et al., 2020a). PRRSV infection has also been associated with alteration in the structure and physiology of the maternal fetal interface, however, it is not known whether such effects are the cause or consequence of fetal death (Barrera-Zarate et al., 2022; Guidoni et al., 2022). Thus, the fundamental mechanism by which transplacental infection results in fetal compromise has yet to be identified, potentially hampering the development of beneficial treatments.

To better understand viral load as it relates to fetal compromise, we first evaluated PRRSV susceptibility of late gestation fetal tissues by assessing the presence of cells expressing the virus’s obligate receptor CD163. We then quantified PRRSV viral RNA in tissues from fetuses classified using an established model of phenotypic extremes in serum and thymic viral load and verified the presence of infectious particles using *in vitro* culture. Finally, we evaluated the impact of viral infection on the local immune response by quantifying IFNG and CD163 expression.

## 2 Materials and methods

### 2.1 Fetal tissues

For assessment of healthy fetal tissues, three Landrace x Large White gilts were selected and housed at the Animal Sciences Research and Education Center (ASREC) at Purdue University in compliance with Institutional Animal Care and Use Committee regulations and approved by the Purdue Animal Care and Use Committee (Protocol #2103002122). The gilts were estrus synchronized using oral progestogen [15 mg/day Altrenogest] and bred via artificial insemination at the first standing estrus post withdraw, and every 24 h after until the end of standing estrus. Pregnancy was verified by ultrasound between days 25 and 30 and gestation and allowed to progress until day 86 relative to the first insemination. Gilts were stunned with a penetrating captive bolt followed by rapid exsanguination to allow for sampling of fetal tissues including the brain (BRN), heart (HRT), liver (LVR), lung (LNG), kidney (KID), and longissimus dorsi muscle (MUS) from healthy viable fetuses. To investigate the distribution of virus in fetal tissues, archived samples were selected from a previously described PRRSV challenge trial (Ko et al., 2022), carried out in strict accordance with the guidelines of the Canadian Council of Animal Care and with approval of the University of Saskatchewan’s Animal Research Ethics Board (Protocol #20180071). In short, N = 22 pregnant gilts (Fast Genetics, Spiritwood, Canada) were inoculated with 1×10^5^ TCID50 PRRSV species 2 (PRRSV-2) strain NVSL 97-7895 on gestation day 86, delivered via a combination of intramuscular injection and intranasal atomization. An additional *N* = 5 gilts were sham inoculated to produce gestationally age matched controls (CON). At 21 days post inoculation (DPI), all animals were humanely euthanized by cranial captive bolt and intravenous barbiturate overdose. The gravid uterus was extracted and the fetal preservation (viable, meconium stained, dead, autolyzed and mummified) and phenotypes, including body weight and crown rump length, assessed as previously described (Ko et al., 2022). Tissue samples were then collected from all fetuses with visible pulsations within the umbilical cord (fetal preservation of viable or meconium stained). Developmentally normal fetuses were further subdivided by degree of fetal preservation, based on the presence and severity of meconium staining. In the absence of meconium staining, fetuses were classified as viable (VIA), while those exhibiting severe meconium staining (head and body) were classified as non-viable (MEC). Fetuses were rapidly exsanguinated post-mortem by severing the axillary artery, and blood samples collected for the isolation of serum. Fetal tissues including the BRN, HRT, LVR, LNG, KID and MUS, along with a combination of cervical and thoracic thymus, were collected from each fetus and individually snap frozen in liquid nitrogen. All samples were stored at-80°C for later analysis. Viral load in fetal serum and thymus was quantified by qPCR as previously described (Malgarin et al., 2021) and used in combination with meconium staining status to identify fetuses in three biologically extreme groups (Table 1). Viable fetuses with no detectable viral load in either serum or thymus were classified as uninfected (UNIF). Fetuses with >5 log_10_ of virus in serum and thymus were considered highly infected, and further subdivided into high viral load viable (HV-VIA), or high viral load meconium stained (HV-MEC). A fourth control group (CON) of viable fetuses was selected from sham inoculated and gestationally age matched gilts. From each of these groups, *N* = 10 fetuses with the lowest z-scores for serum T4 were selected as previously described, such that no significant differences in other phenotypic parameters such as fetal weight or crown rump length existed between groups (Mulligan et al., 2022).

**Table 1:** Phenotypes of fetal groups representing biological extremes in resistance in response to maternal PRRSV infection.

				Viral load^*^	
Group	*N*	Fetal weight (g)	Crown rump length (cm)	Serum	Thymus
CON	10	990.77 ± 218.76	35.7 ± 2.5	0 (0–0)	0 (0–0)
UNIF	10	956.13 ± 217.78	36.5 2.8	0 (0–0)	0 (0–0)
HV-VIA	10	907.40 ± 216.55	35.9 ± 2.9	7.79 (5.93–9.22)	6.73 (5.51–7.89)
HV-MEC	10	1044.7 ± 249.78	35.7 ± 3.1	8.22 (7.41–9.09)	6.74 (5.01–8.09)

Fetal weight and crown rump length are reported as mean ± standard deviation.

^*^serum and thymic viral load in the form of log 10 RNA copies per mg (thymus) or mL (serum) and reported as mean (range).

### 2.2 Immunohistofluorescence

Samples of BRN, HRT, LVR, LNG, KID, and MUS from healthy fetuses, derived from unchallenged gilts at day 86 of gestation were suspended in optimal cutting temperature media (OCT) and frozen on the surface of a dry ice block. Sequential 10 μm thick cross-sections of each tissue were cut on a Leica CM1950 cryotome at −20°C, affixed to Superfrost Plus slides (Thermo Fisher Scientific, Waltham, MA, United States) and air-dried for 30 min at room temperature. Slides were rehydrated in phosphate buffered saline (PBS) before fixing in ice-cold acetone for 10 min. Tissue sections were blocked against non-specific binding for 2 h with 0.1% w/v BSA and 10% horse serum in PBS. Sections were then incubated over night at 4°C with 10 μg/mL mouse anti-bovine CD163 [Clone LND68A, Washington State Monoclonal Antibody Center], previously shown to cross-react with both isoforms of the porcine target (Pasternak et al., 2019), or the equivalent concentration of mouse IgG1 isotype control. Sections were then washed three times in PBS before incubating with 1 μg/mL Alexa Fluor 555 conjugated F(ab’)2-goat anti-mouse IgG, secondary antibody for 2 h at room temperature. Slides were again washed three times before counter staining with 0.5 μg/mL DAPI in methanol for 10 min at room temperature and cover slipping with Mowiol. Fluorescent evaluation and imaging were carried out in triplicate using an Echo Revolution microscope equipped with 10× and 20× objectives.

### 2.3 Viral load and host gene expression in non-lymphoid tissues

Samples of BRN, HRT, LVR, LNG, KID and MUS from each selected fetus were cryogenically homogenized using a pre-chilled mortar and pestle. Trizol (Thermofisher, Waltham, MA, United States) was then used to extract total RNA from a subsample of each tissue homogenate using a double precipitation protocol (Oliver et al., 2011). DNA contamination was removed using the Turbo DNA-free Kit (Invitrogen) as per the manufacturer’s instructions, but with the addition of 5 U of RNase Inhibitor (ThermoFisher). Concentration and purity of the RNA was assessed using a Nanodrop ND-1000 spectrophotometer (ThermoFisher), and integrity was evaluated using denaturing gel electrophoresis (Kent-Dennis et al., 2019). Reverse transcription with 2 μg total RNA was carried out using the High-Capacity cDNA Reverse Transcription Kit (Applied Biosystems, Foster City, CA, United States). Absolute quantification of PRRSV-2 was carried out using virus specific primers (5′-TAATGGG CTGGCATTCCT-3′ and 5′-ACACGGTCGCCCTAATTG-3′) and a corresponding Taqman probe (5′-FAM-TGTGGTGAATG GCACTGATTG-BHQ-3′), with a seven-point standard curve starting at 10^7^ copies/μL of the target amplicon cloned into TOPO vector (Invitrogen, Carlsbad, CA, United States). Host gene expression was measured using previously validated gene specific primers (Table 2). All qPCR assays were carried out in duplicate using 20 ng cDNA per reaction on a CFX Connect Real-Time System (Bio-Rad) using SsoAdvanced Universal SYBR Green SuperMix (Bio-Rad). Absolute quantification for viral load is expressed in the form of copy number (CN) per 20 ng equivalent cDNA as previously established (Ison et al., 2022). For relative quantification, the stability of eight housekeeping genes was evaluated, and the geometric mean of two or three stable genes in each tissue was used to normalize expression of each gene of interest, and fold change was then calculated relative to the mean expression in the control group using the 2^−ΔΔCT^ method.

**Table 2:** Porcine specific qPCR primer sequences.

Target	NCBI Gene ID	Forward primer	Reverse primer	Tm (°C)	Length (bp)	Target sequence or Reference
ACTB	414,396	5′-CCAGCACGATGAAGATCAAG-3′	5′-AGTCCGCCTAGAAGCATTTG-3′	60	171	Ison et al. (2023)
CD163	397,031	5′-ATTACCTGCTCAGCCCACAG-3′	5′-CGCCTCCAGAGAGAAGTCAG-3′	61	126	NM_213976.1
HMBS	396,581	5′-AGGATGGGCAACTCTACCTG-3′	5′-GATGGTGGCCTGCATAGTCT-3′	61	83	Pasternak et al. (2016)
IFNG	396,991	5′-GCTCTGGGAAACTGAATGAC-3′	5′-TCTCTGGCCTTGGAACATAG-3′	61	167	Pasternak et al. (2020a)
PPIA	397,637	5′-CACTGCCAAGACTGAGTGGT-3′	5′-TGTCCACAGTCAGCAATGGT-3′	61	144	Pasternak et al. (2020b)
RPL19	396,989	5′-AACTCCCGTCAGCAGATCC-3′	5′-AGTACCCTTCCGCTTACCG-3′	60	147	Pasternak et al. (2015)
SDHA	780,433	5′-CTACAAGGGGCAGGTTCTGA-3′	5′-AAGACAACGAGGTCCAGGAG-3′	61	141	Pasternak et al. (2016)
STX5	100,628,048	5′-TGCAGAGTCGTCAGAATGGA-3′	5′-CCAGGATTGTCAGCTTCTCC-3′	60	144	Pasternak et al. (2020a)
TBP	110,259,740	5′-CTGAATGCTGAGGCGATTTC-3′	5′-GCTGTGGAGTCAGTCCTGTG-3′	61	186	Pasternak et al. (2020b)
YWHAZ	780,440	5′-TGATGATAAGAAAGGGATTGTGG-3′	5′-GTTCAGCAATGGCTTCATCA-3′	62	203	Pasternak et al. (2020a,b)

### 2.4 Viral recovery from tissue

MARC-145 cells were cultured in high glucose minimal essential media supplemented with 10% fetal bovine serum, 100 units/mL of penicillin, 100 μg/mL of streptomycin, and 0.225 μg/mL of amphotericin B. Where sufficient sample of fetal tissue were available, 70–100 mg aliquots, previously ground to a fine powder under liquid nitrogen, were mixed with culture media at a ratio of 1 mg/mL. Zirconia beads (1 mm, biospec) were added, and the mixture further homogenized for two 1 min periods at 60 Hz. The mixture was then centrifuged at 10,000 × g for 10 min and the resulting lysate collected. MARC-145 cells in 96 well plates were inoculated in triplicate with 100 μL of tissue lysate and cultured for 72 h at 37°C and 5% CO_2_. Media was aspirated and the cells washed twice with PBS, followed by subsequent fixation with 80% acetone for 10 min. The acetone was then removed and the plates dried for 1 h at room temperature. Cells were then blocked with 1% BSA in PBS, before incubating with a 1:1000 dilution of mouse anti-PRRSV antibody (SDOW-17, RTI, Brookings, SD) for 30 min at 37°C. Cells were then washed twice with PBS before incubating with 2 μg/mL Alexa488 conjugated goat anti mouse IgG1 (A-21121, Thermofisher scientific) for 15 min at room temperature. Monolayers were then washed twice with PBS and counter stained with 1 μg/mL DAPI for 5 min at room temperature, washed with PBS, and then examined for PRRSV infection on an inverted fluorescent scope. Virus was deemed recovered from a given sample if positive PRRSV staining was observed in one or more wells.

### 2.5 Data analysis

All data processing and statistical analyses were performed in R 4.2.3 (R Core Team, 2019). Viral load and host tissue gene expression data were assessed for normality using a combination of graphical assessment and Shapiro-Wilks test and found to be non-normal. Data was subsequently evaluated within tissue using a non-parametric approach consisting of Kruskal-Wallis followed by a pairwise Wilcox test with Benjamini-Hochberg correction for multiple group testing. Numerical data was visualized using the ggplot2 package (Wickham, 2016) with statistically significant differences (*p* < 0.05), where present, marked with unique superscripts.

## 3 Results

### 3.1 CD163 in late gestation fetal tissues

**Figure 1:**
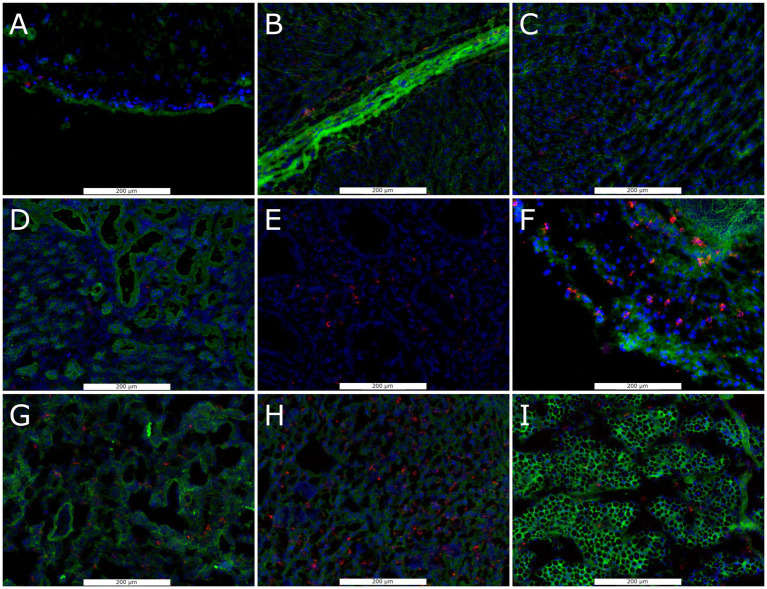
Representative florescent images of CD163+ cell staining in frozen sections of non-lymphoid fetal tissues collected at day 86 of gestation, including brain **(A)**, heart including epicardium **(B)**, and myocardium **(C)**, kidney including cortex **(D)**, collecting duct **(E)**, and capsule **(F)**, lung **(G)**, liver **(H)** and loin muscle **(I)**. Sections were stained with antibodies against anti-CD163 (Red), with DAPI (blue) and wheat germ agglutinin (Green) as a counter stains.

To determine the capacity for PRRSV-2 infection in late gestation non-lymphoid fetal tissues, we assessed the availability of the obligate receptor, CD163, by immunohistoflourescence and carried out a qualitative assessment of morphology and distribution. Positive staining for CD163 was observed on the surface of cells in all six non-lymphoid tissues from healthy day 86 fetuses, though the relative density, morphology and spatial localization of positive cells varied between tissue (Figure 1). In the brain, staining for CD163 found positive cells to be spatially restricted to the pia matter (outer most surface of the BRN sections evaluated). In the HRT, clusters of positive cells were observed throughout the ventricular myocardium and epicardium. Positive cells were observed throughout the KID but appeared to be enriched within the collecting ducts and renal capsule relative to the cortex. In the LNG, positive staining was observed within the interstitial compartment and alveolar spaces. In the LVR, positive CD163 staining of small cells were found to be evenly distributed throughout the hepatic lobule. Positive staining of satellite cells, closely associated with the surface of muscle fibers were observed throughout the cross section of MUS.

### 3.2 Viral load in non-lymphoid tissues

**Figure 2:**
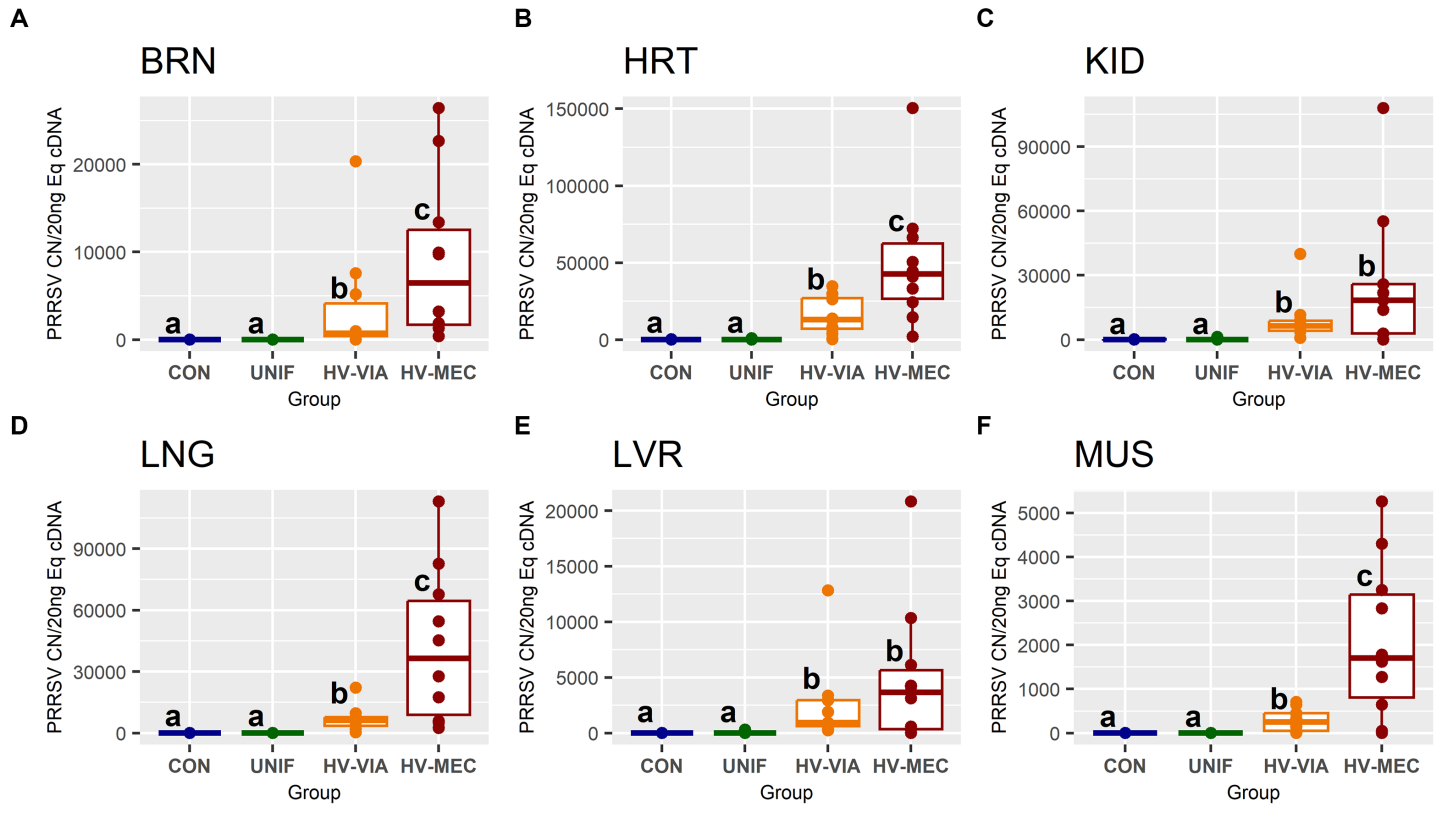
Viral RNA, as measured by PRRSV-2 strain specific absolute quantification qPCR, in fetal brain (BRN), heart (HRT), kidney (KID), lung (LNG), liver (LVR) and skeletal muscle (MUS) in fetuses uninfected (UNIF), high viral load viable (HV-VIA) and high viral load meconium stained (HV-MEC), 21 days after maternal PRRSV2 challenge, along with equivalent tissues from control (CON) fetuses derived from gestationally age matched and sham inoculated dams. Viral load is expressed as copy number (CN) per 20 ng equivalent cDNA, with unique superscripts indicating statistically significant differences (*p* < 0.05).

To evaluate the broader distribution of PRRSV-2 RNA, we initially assessed viral load in six non-lymphoid tissues including BRN, HRT, KID, LNG, LVR and MUS (Figure 2) in fetuses from PRRSV-2 infected gilts. No significant viral load was observed in any of the six tissues in fetuses previously categorized at UNIF based on prior assessment of serum and thymus. In contrast, significant quantities of viral RNA were detected in all six tissues derived from high infected fetuses. Overall, the median viral load for HV fetuses was greatest in the HRT. The highest viral load in non-lymphoid tissues was also detected in the HRT (x̃ = 26,734 CN/20 ng cDNA), and significantly greater (*p* = 0.021) virus concentration was found in HRT tissue from HV-MEC (x̃ = 42,798 CN/20 ng cDNA) relative to HV-VIA (x̃ = 13,233 CN/20 ng cDNA). A similar median viral load was detected in KID and LNG from HV fetuses (x̃ = 7,445 and x̃ = 7,779 CN/20 ng cDNA respectively). While a significant difference (*p* = 0.037) was identified in the LNG of HV-VIA (x̃ = 6,207 CN/20 ng cDNA) compared to HV-MEC (x̃ = 36,534 CN/20 ng cDNA), no significant difference (*p* = 0.41) between HV-VIA (x̃ = 6,426 CN/20 ng cDNA) and HV-MEC (x̃ = 18,444 CN/20 ng cDNA) was observed in KID. Equivalent median viral loads were also observed in BRN and LVR (x̃ = 1,774 and x̃ = 1,900 CN/20 ng cDNA respectively). Viral load was significantly (*p* = 0.045) greater in BRN tissues from HV-MEC (x̃ = 6,486 CN/20 ng cDNA) than HV-VIA (x̃ = 743 CN/20 ng cDNA), but equivalent in LVR of HV-VIA (x̃ = 950 CN/20 ng cDNA) and HV-MEC (x̃ = 3,658 CN/20 ng cDNA) fetuses. The lowest viral load in tissues from HV fetuses was detected in MUS (x̃ = 548 CN/20 ng cDNA), with significantly (*p* = 0.02) greater virus observed in MUS from HV-MEC (x̃ = 1,704 CN/20 ng cDNA) relative to HV-VIA (x̃ = 256 CN/20 ng cDNA) fetuses.

### 3.3 Viral recovery

To confirm the presence of infectious viral particles, we inoculated MARC-145 cells with fetal tissue lysates and evaluated viral recovery via immunofluorescent staining with a PRRSV specific monoclonal antibody (Table 3). No virus was recovered from lysates prepared from any of the six tissues isolated from control fetuses. In contrast, virus was successfully recovered in over 90% in HRT, LNG and MUS samples originating from highly infected (HV-VIA & HV-MEC) fetuses. Overall viral recovery rates were lower in KID, LVR and BRN, at 70, 42 and 30%, respectively. No significant difference in the recovery rate was observed between HV-VIA and HV-MEC fetuses for any of the tissues evaluated.

**Table 3:** Rate of viral recovery using cell lysates from 6 non-lymphoid tissues derived from high viral load viable (HV-VIA) and meconium stained (HV-MEC) fetuses.

	Fetal classification			
Tissue	Control	HV-VIA	HV-MEC	Fisher’s *p*-value^*^
Brain	0/3 (0%)	4/10 (40%)	2/10 (20%)	0.629
Heart	0/3 (0%)	8/9 (88.89%)	17/17 (100%)	0.346
Kidney	0/3 (0%)	11/16 (68.75%)	13/18 (72.22%)	1
Liver	0/4 (0%)	4/10 (40%)	4/9 (44.44%)	1
Lung	0/3 (0%)	8/9 (88.89%)	10/10 (100%)	0.474
Muscle	0/4 (0%)	9/10 (90%)	8/8 (100%)	1

^*^p-value for Fisher exact test for recovery rate between HV-VIA and HV-MEC groups.

### 3.4 Impact of local infection

**Figure 3:**
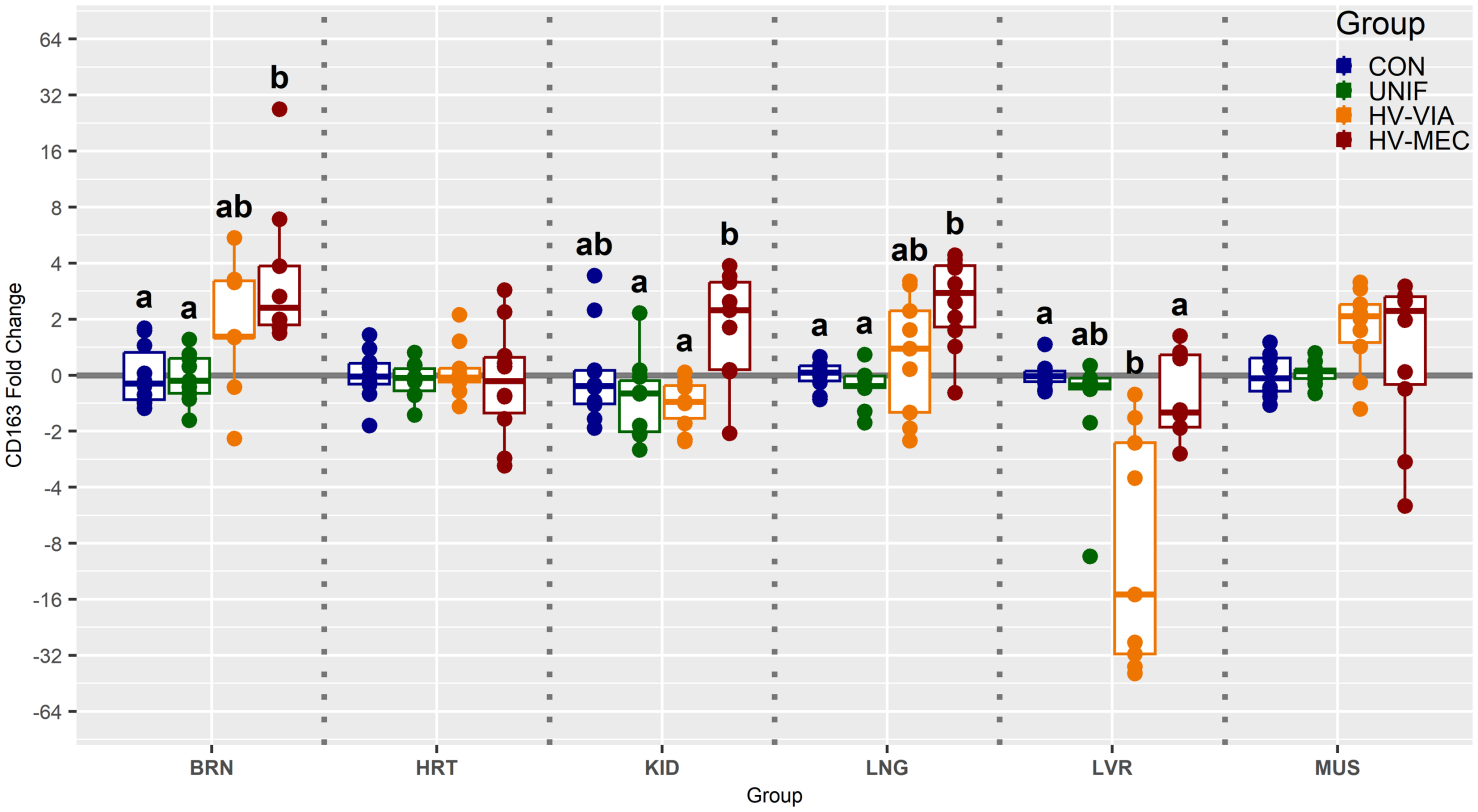
Expression of cluster of differentiation marker 163 (CD163) as measured by qPCR in fetal brain (BRN), heart (HRT), kidney (KID), lung (LNG), liver (LVR) and skeletal muscle (MUS) in fetuses uninfected (UNIF), high viral load viable (HV-VIA) and high viral load meconium stained (HV-MEC), 21 days after maternal PRRSV2 challenge, along with equivalent tissues from control (CON) fetuses derived from gestationally age matched and sham inoculated dams. Fold changes are calculated within tissue relative to the average expression of in the control group with unique superscripts indicating statistically significant differences (*p* < 0.05).

**Figure 4:**
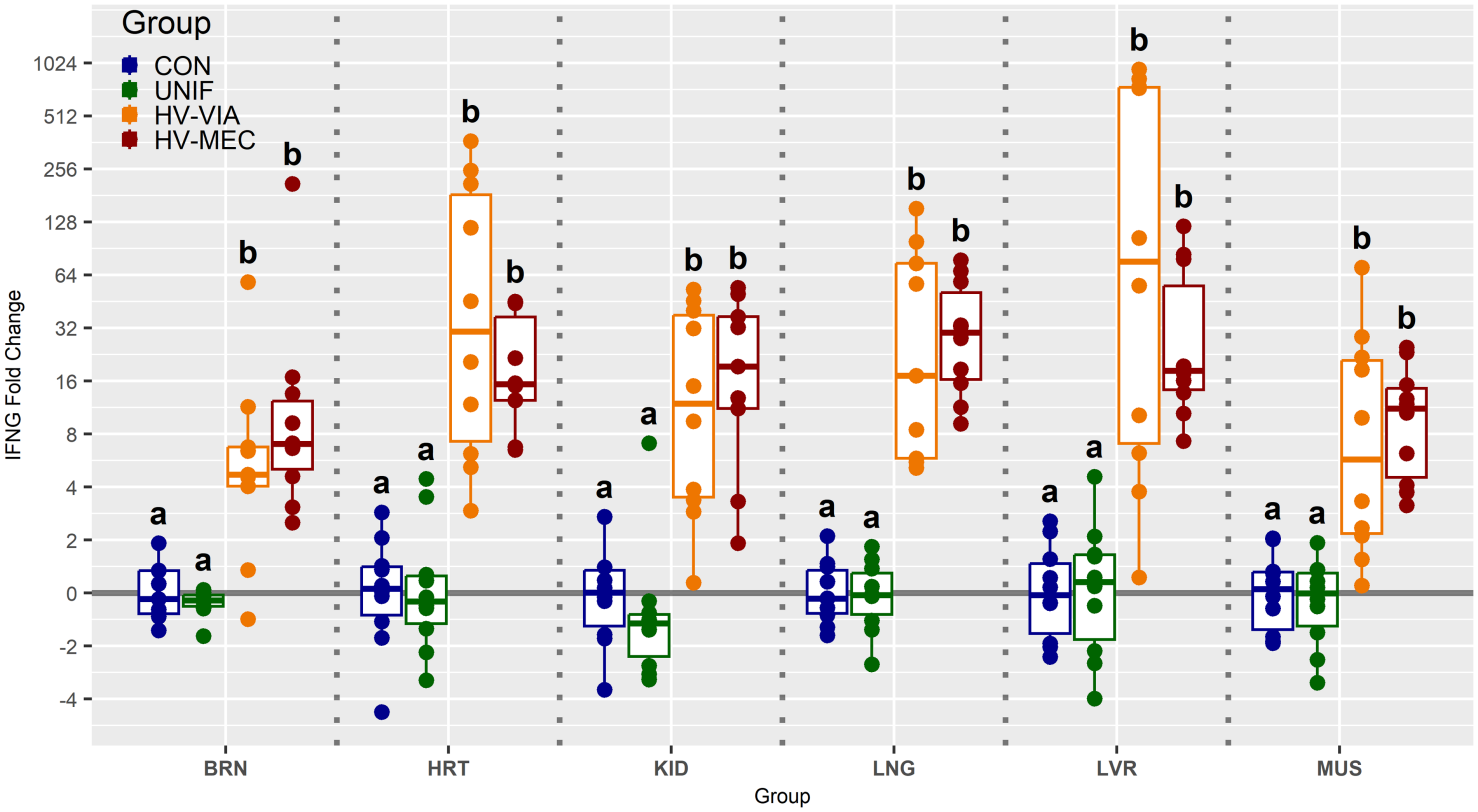
Expression of interferon gamma (IFNG) as measured by qPCR in fetal brain (BRN), heart (HRT), kidney (KID), lung (LNG), liver (LVR) and skeletal muscle (MUS) in fetuses uninfected (UNIF), high viral load viable (HV-VIA) and high viral load meconium stained (HV-MEC), 21 days after maternal PRRSV2 challenge, along with equivalent tissues from control (CON) fetuses derived from gestationally age matched and sham inoculated dams. Fold changes are calculated within tissue relative to the average expression of in the control group with unique superscripts indicating statistically significant differences (*p* < 0.05).

To determine if viral infection status impacts the local population of susceptible cells, we evaluated the gene expression of CD163 across six non-lymphoid fetal tissues from PRRSV infected gilts (Figure 3). Median expression of CD163 was significantly increased by 2.3 fold in the BRN (*p* < 0.001), 2.7 fold in the LNG (*p* = 0.005) and 2.2 fold in the KID (*p* = 0.19) of HV-MEC fetuses relative to UNIF. Increased expression of CD163 in MUS from HV-MEC fetuses showed a trend toward significance (*p* = 0.052) relative to both CON and UNIF groups. In contrast, gene expression of CD163 in the LVR of HV-VIA fetuses was decreased by 15 fold relative to CON (*p* = 0.002). No significant change in CD163 was observed in the HRT. Finally, to understand the local inflammatory response following infection of non-lymphoid tissues, we evaluated gene expression of IFNG (Figure 4). No significant difference in IFNG expression was observed any of the six tissues from UNIF fetuses relative to CON. Expression was significantly increased relative to CON in all six tissues from HV-VIA and HV-MEC fetuses, but no significant difference between these two highly infected groups was observed. The largest increases in median IFNG gene expression among highly infected fetuses were found in the HRT (x̃ = 18.1 fold), KID (x̃ = 15.0 fold), LNG (x̃ = 27.9 fold) and LVR (x̃ = 19.2 fold), while upregulation in the MUS (x̃ = 10.2 fold) and BRN (x̃ = 6.6 fold) were comparatively lower.

## 4 Discussion

PRRSV is capable of crossing the late gestation porcine placenta and productively infecting the fetus. Unlike postnatal animals, where the primary site of infection is the lung, the thymus has historically been recognized as the primary site of viral replication in the fetus (Rowland, 2010). Assessment of viral load in additional samples including blood and placenta have been used in conjunction with thymus to categorize fetal infection status and evaluate progressive severity in infection (Van Goor et al., 2020). While such studies have evaluated the physiological impact of fetal systems logically associated with viability such as immune regulation (Pasternak et al., 2020b), cardiovascular stress (Pasternak et al., 2020a; Malgarin et al., 2021), and placental function (Guidoni et al., 2021; Barrera-Zarate et al., 2022), no definitive explanation for virus-induced fetal compromise has been identified. However, a number of studies have found evidence to suggest infection of other fetal organs does occur (Cheon and Chae, 2000; Cheon and Chae, 2001; Rowland, 2010), although sporadically within the larger fetal population. Thus, the objective of the present study was to evaluate the capacity for PRRSV infection in non-lymphoid organs, their response to infection and the association with fetal viability.

Cellular infection by PRRSV is dependent on the presence of its obligate receptor CD163 (Van Gorp et al., 2008), which under normal circumstances functions in the clearance of hemoglobin (Kristiansen et al., 2001). This cell surface protein is primarily found on monocytes, macrophages (Buechler et al., 2000) and specific subsets of dendritic cells (Maniecki et al., 2006). The majority of postnatal tissues contain a resident population of macrophages which are established during early embryonic development, though the equivalent population in a subset of tissues is known to be generated postnatally from blood derived macrophages (Davies et al., 2020). Regardless of their source, most subpopulations of mature tissue macrophages express the hemoglobin-haptoglobin scavenger CD163 (Fabriek et al., 2005). Previous work in fetal pigs has demonstrated the presence of hepatic, splenic and pulmonary CD163 positive cells throughout gestation (Karniychuk and Nauwynck, 2009), but the presence of such cells in other fetal tissues has not been previously established. Using a similar Immunohistofluorescence approach, we have demonstrated the presence of CD163 positive cells in four additional fetal organs including HRT, KID, BRN and MUS at day 86 of gestation, indicating widespread capacity for PRRSV infection across non-lymphoid tissues.

Interestingly, the apparent abundance, spatial distribution and morphology of CD163 cells varied substantially between tissues. In the LVR, CD163 staining was associated with relatively small cells with even distribution throughout the tissue, which would be consistent with Kupffer cells. As with previous reports in both humans (Naito et al., 1997) and swine (Karniychuk and Nauwynck, 2009), a large number of such cells were observed evenly distributed throughout the fetal LVR. Also consistent with previous reports (Karniychuk and Nauwynck, 2009), the abundance of cells positively stained for CD163 was comparatively low in pulmonary tissues. Macrophages are the most abundant immune cell within the heart, a population which is established during early embryonic development by yolk sac derived progenitors (Epelman et al., 2014). It is thus unsurprising that a substantial population of CD163 positive staining was observed in cardiac tissue from the late gestation fetal pig, where it was associated with relatively large cells within the tissue of the ventricle. Cells with similar morphology were found throughout the fetal pig KID, though a substantial enrichment was observed in remnants of the renal capsule. Staining in the skeletal muscle was associated with small cells on the surface of muscle fibers, consistent with muscle resident macrophages known to express high levels of CD163 and originate from both embryonic yolk sac and liver, as well as postnatally from blood monocytes (Wang et al., 2020). In the brain, positive staining for CD163 was largely restricted to the pia mater, which is known to contain border associated macrophages (Taketomi and Tsuruta, 2023). The apparent lack of staining in the neural parenchyma is consistent with prior observations which show an absence of macrophages in the cerebrum and cerebellum during late gestation (Matsumoto and Ikuta, 1985).

Given these resting populations of PRRSV susceptible resident cells during late gestation, it is perhaps unsurprising that viral RNA was detected in samples of all six non-lymphoid tissues from fetuses previously classified as high viral load based on serum and thymus alone. The presence of infectious viral particles in these tissues is further supported by high success rates for *in vitro* recovery from the corresponding tissue lysates. Comparatively high viral load in the HRT relative to the LNG is consistent with prior work by other labs investigating late gestation fetal infection (Rowland, 2010). By definition, serum from HV-VIA and HV-MEC fetuses contained greater than 10^5^ copies of viral RNA per μl, and thus the contribution of virus from circulation to the present observations in tissues cannot be ruled out. Fetuses in the present trial were exsanguinated via the axillary artery, and while the efficacy of this approach in reducing blood volume in various fetal organs has not been established, when employed postnatally for the purpose of slaughter, total blood volume is only reduced by 40%–60%, but residual content in lean meat reduced to just 2–9 mL/kg (Warriss, 1984). In addition, studies investigating the presence of virus in stillborn PRRSV infected piglets using immunohistochemistry have shown positive viral staining in both cardiac and renal tissue (Cheon and Chae, 2000; Cheon and Chae, 2001).

More critically, while fetuses identified as HV-VIA and HV-MEC have equivalent viral load in serum and thymus (Table 1), the viral load measured by qPCR in BRN, HRT, LNG and MUS was significantly elevated in HV-MEC fetuses compared to HV-VIA. This result may indicate that increased infection of critical non-lymphoid organs may be a causative factor differentiating between resilient and susceptible fetuses. Elevated viral load in the heart is of specific interest with regards to explaining fetal compromise. In humans, *in utero* infection with viral pathogens such as rubella has been associated with altered cardiac developmental processes and structural defects (Singampalli et al., 2021). Elevated viral load in the brain is of similar interest, given prior observations with Zika virus, which has been show to infect the fetal brain (Retallack et al., 2016) and result in abnormalities including microcephaly and, in some cases, fetal death (Leisher et al., 2021).

In conjunction with infection, we observed elevated expression of CD163 in the BRN, KID and LNG of HV-MEC fetuses which could be explained by three distinct hypotheses. The first of which is transcriptional upregulation in the tissue resident macrophages, which occurs during the establishment of an activated phenotype (Etzerodt and Moestrup, 2013). Second is that tissue resident macrophages are able to re-enter the cell cycle despite being terminally differentiated (Kulle et al., 2022), so the apparent upregulation could indicate proliferation resulting in a proportional increase of CD163+ cells relative to other constituents. Finally, increased transcription of CD163 could be associated with recruitment of blood monocytes or macrophages from other tissues. As the liver is the original source from which other tissue resident macrophage pools are created (Hoeffel and Ginhoux, 2015), this latter hypothesis may be supported by the decrease in hepatic CD163. The fact that this change in hepatic expression occurs in the HV-VIA rather than HV-MEC may simply indicate an early stage in the migration process. The observed stability of cardiac CD163 expression across phenotypic groups, in conjunction with significant viral load in the highly infected fetuses, suggests that the population of susceptible cells was present prior to infection and is not the product of macrophage infiltration.

Even in the absence of standard clinical signs, maternal infection with PRRSV produces a robust immune response characterized by increases in circulating IFNα and IFNγ (Ladinig et al., 2015). Following vertical transmission, the fetus displays a similar increase in serum type 1 and 2 interferons (Pasternak et al., 2020b). This is largely consistent with the local immune responses within fetal lymphoid organs such as the spleen and thymus, where there is a robust upregulation in expression of IFNB, IFNG and an array of other cytokines and chemokines (Pasternak et al., 2020a,b). The observed increase in IFNG expression in the fetal brain and lung observed in the present study are consistent with prior work from other investigators (Rowland, 2010; Antonson et al., 2018). The present study is, however, the first to demonstrate a local immune response, in the form of upregulated IFNG, within cardiac, hepatic, renal and musculoskeletal tissues. Due to the timing of sample collection relative to maternal challenge, it is not possible to differentiate between IFNG produced by resident cells present in the tissues prior to infection and those with which may have infiltrated following infection, however, regardless of the cellular source, the increased IFNG expression following infection indicates a localized inflammatory response. The observed upregulation in IFNG within these six non-lymphoid tissues was entirely restricted to highly infected fetuses, further supporting local viral infection. Interestingly, there was no difference in immune response between HV-VIA and HV-MEC groups, indicating the local immune interferon response is not associated with loss of fetal viability.

## 5 Conclusion

Collectively, our results show that non-lymphoid fetal organs not only contain a population of PRRSV susceptible cells, but harbor infectious viral particles following vertical PRRSV transmission. Most critically, the concentration of viral RNA within fetal HRT, BRN, LNG and MUS was elevated in compromised, meconium-stained fetuses compared to infected viable fetuses. Both local infection and interferon expression were found to be coincident in all tissues, though the latter was not associated with loss of fetal viability. Further evaluation of the acute physiological effects of infection and inflammation in these non-lymphoid tissues will be critical in understanding the mechanism by which *in utero* PRRSV infection compromises fetal viability. In addition, fetal PRRSV infection may represent a high value biomedical model to study the long term developmental effects of *in utero* inflammation.

## Data availability statement

The datasets presented in this study can be found in online repositories. The names of the repository/repositories and accession number(s) can be found at: https://github.com/JAlexPasternak/Fetal-_PRRSV_Distribution.

## Ethics statement

The animal study was approved by Purdue University Institutional Animal Care and Use Committee and University of Saskatchewan’s Animal Research Ethics Board. The study was conducted in accordance with the local legislation and institutional requirements.

## Author contributions

KR: Investigation, Methodology, Validation, Writing – review & editing. DJ: Investigation, Methodology, Validation, Writing – review & editing. AS: Investigation, Methodology, Validation, Writing – review & editing. JH: Conceptualization, Funding acquisition, Methodology, Project administration, Writing – review & editing. JP: Conceptualization, Data curation, Formal Analysis, Funding acquisition, Investigation, Methodology, Project administration, Resources, Software, Supervision, Validation, Visualization, Writing – original draft, Writing – review & editing.
